# Supplementary material for: Distinct cellular states determine calcium signaling response
Source: Mol Syst Biol. 2016 Dec 15;12(12):894. doi: 10.15252/msb.20167137 (PMC5199124; doi:10.15252/msb.20167137)
Supplement: Supplementary file 1 — Expanded View Figures PDF [file MSB-12-894-s001.pdf]

Expanded View Figures

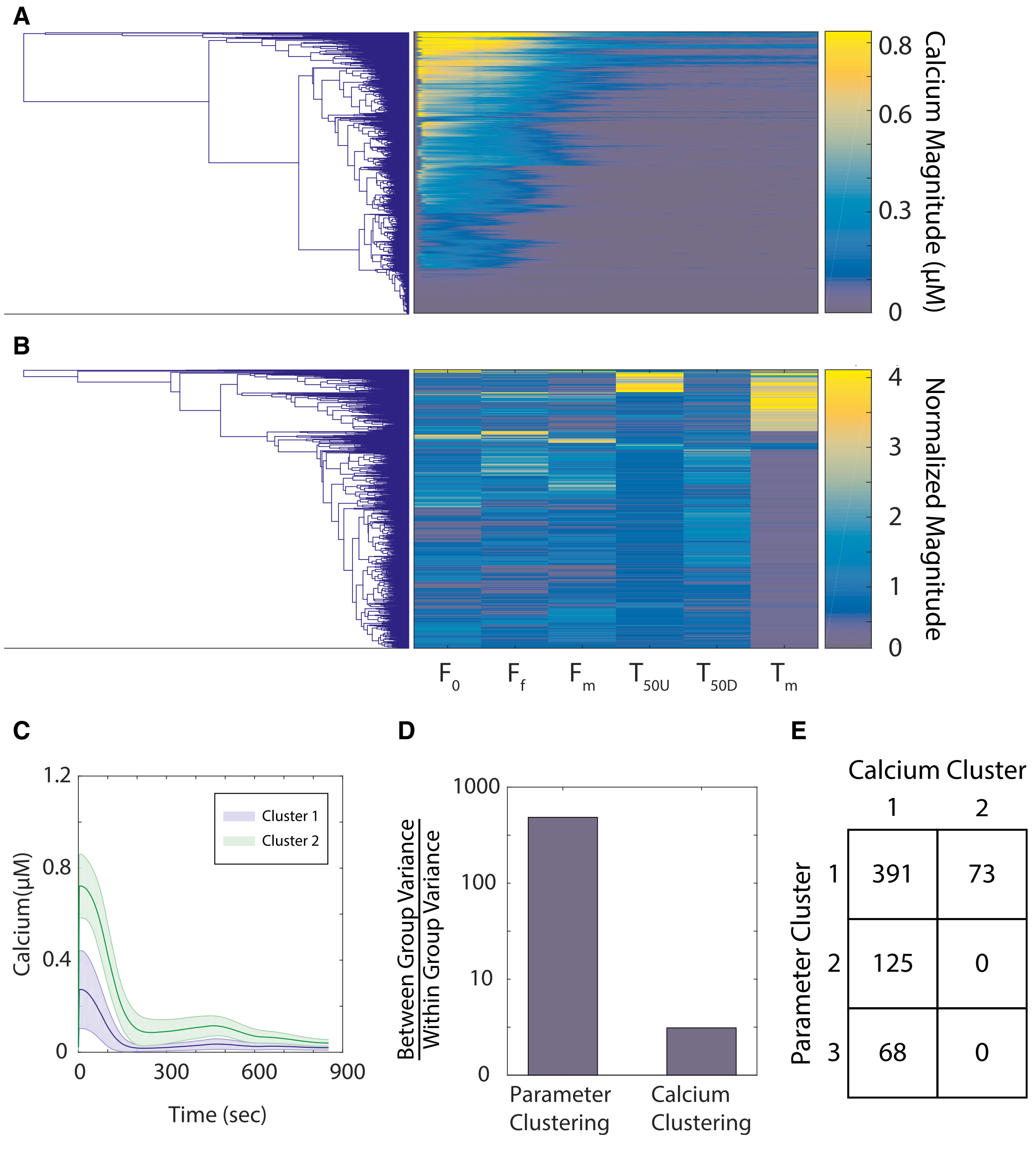

Figure EV1. Clustering results for cells based on only time-course data.

A Clustering based on calcium data. Results of cluster analysis based on the Euclidean distances between the whole time series of calcium data. The left side of the panel shows the dendrogram, and the right side of the panel shows the corresponding matrix of all the time-course calcium data, with each row being from a single cell and the columns being the time points.

B Clustering based on time-series features. The left side of the panel shows dendrogram of the clustering based on time-series features of the data, and the right side of the panel shows the corresponding matrix of the time-series features of the data. Each of the rows represents a single cell; the columns are elements of the time-series features for the cell. The features from left to right are basal level before stimulation, time taken to reach half maximal level, time taken to reach maximum level, maximum level, time from maximal level to half maximal, and steady state after stimulation. The individual features are normalized with respect to the average values.

C The plot of the average and standard deviation of calcium data of the two clusters of the clustering that was based on calcium response data where the hierarchical tree (panel A) was thresholded based on the Calinski–Harabasz criterion.

D The bar graph of the ratio of between group variance to within group variance from both the parameter clustering (Fig 4) and calcium data clustering (panel A). The results show that the kinetic parameter clustering scheme has higher separation between the individual clusters compared to that of calcium data.

E The confusion table of cells that belong in the three most significant identified clusters in parameters and the two clusters with respect to calcium data clusters.

© 2016 The Authors

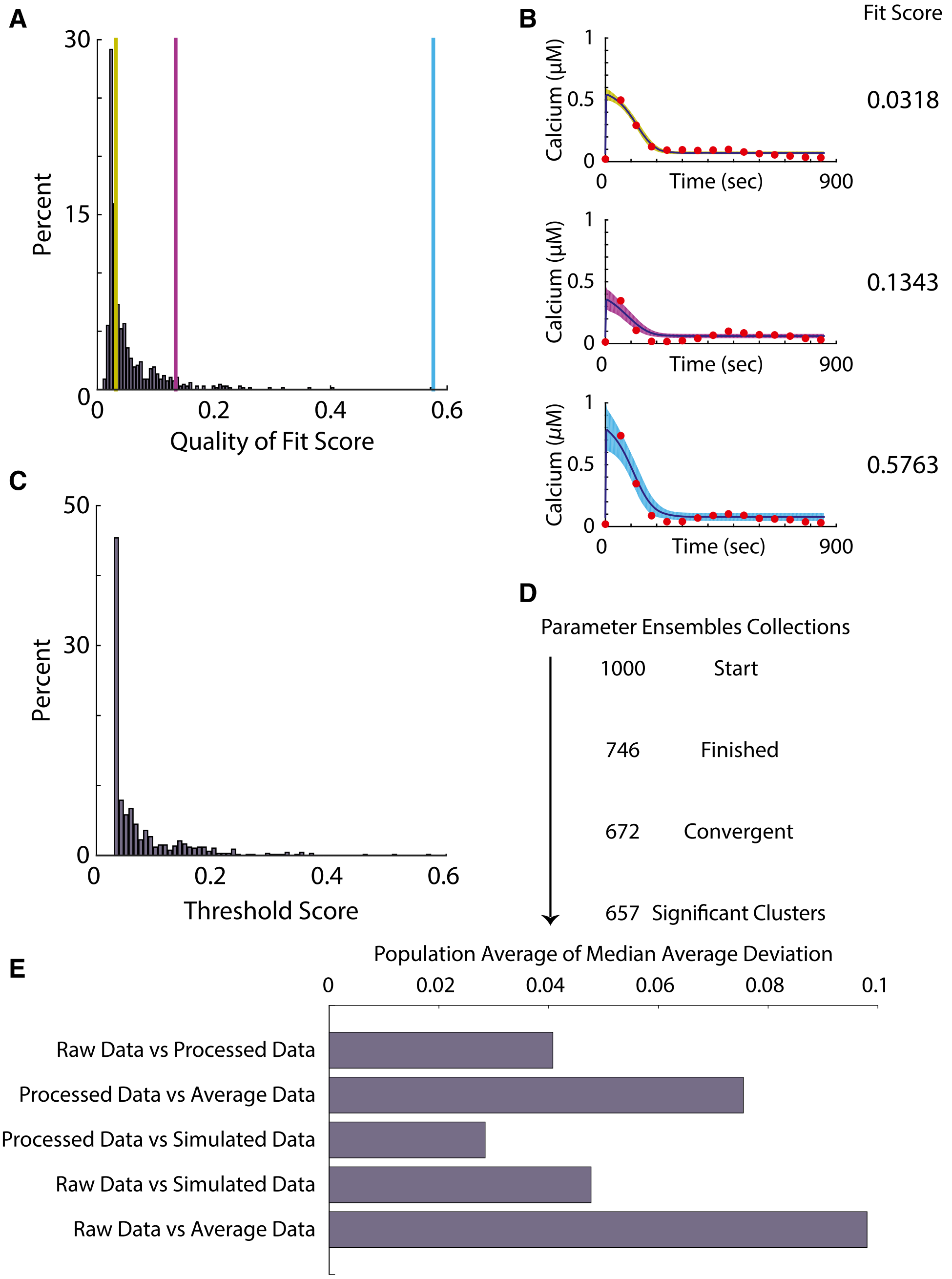

Figure EV2. Quality of convergent single-cell model fitting.

A Histogram of average fit scores of convergent parameters. Each of the single-cell data is fitted to the differential equations model to produce a posterior parameter vector population and the average of the scores of the simulated data is calculated. The histogram shows the distribution of the average goodness-of-fit scores for all cells. The three vertical color bars show examples of three fits shown in panel (B).

B Example Bayesian fit. The three instances of Bayesian fits show the fitted data as red dots, the average of the simulated data in dark blue trajectories, and confidence intervals whose colors correspond to their respective location in the histogram in panel. The fit ensembles average score is shown on the right of each plot. The three examples demonstrate visually the spectrum of quality of fits from the algorithm.

C In our fit, each cell has its own final acceptance threshold based on experimental noise for that cell. The histogram shows the distribution of these acceptance threshold scores. All scores in panel (A) are below the corresponding individualized acceptance value show in (C).

D The progression number of cells in the fitting process. The entire workflow started out with 1,000 fits. Due to technical issues, 746 instances of fitting successfully finished. After the test of convergence, 672 fits were found to be convergent; out of the convergent instances of fits, 657 were found to belong to one of the three major clusters of parameter ensembles.

E The bar plot of the median absolute deviation comparing raw data, processed data, and simulation for each single cell.

 © 2016 The Authors

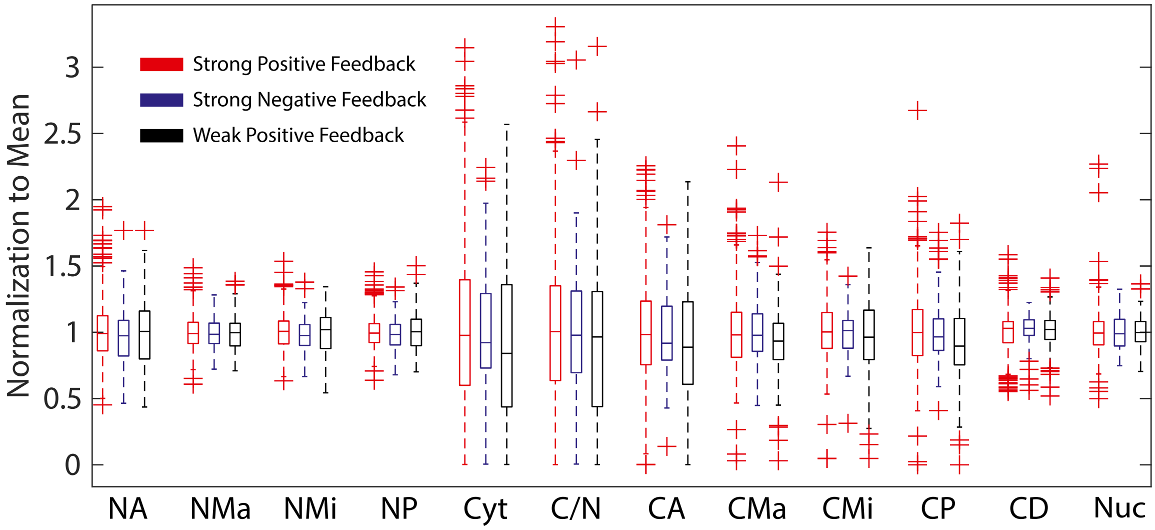

Figure EV3. Distribution of cell physical features among clusters.
The boxplots of the different cell physical features according to the three clusters, normalized with respect to the mean values. The acronyms of the cell physical features are the nuclear area (NA), major axis of nuclei (NMa), minor axis of nuclei (NMi), perimeter of the nuclei (NP), cytoplasm area (Cyt), ratio of cytoplasm to nuclei area (C/N), cytoplasm area (CA), cytoplasm area major axis (CMa), cytoplasm area minor axis (CMi), cell perimeter (CP), effective local cell density (CD), Hoechst intensities (Nuc). The red lines of the boxplots represent the median of the distributions, and the ranges of the whiskers are defined to be 1.5 times the interquartile range (difference between 75th and 25th percentiles). Crosses are data outside of the interquartile range.

Figure EV4. Boxplot of kinetic parameter distributions in the major clusters.

A The 17 boxplots show the variance of the 17 parameters in the three major clusters: strong positive feedback (SP), strong negative feedback (SN), and weak positive feedback (WP).

B The boxplots of the cell calcium data temporal features belonging to the three major clusters. The values are normalized according to the mean. The red lines of the boxplots represent the median of the distributions, and the ranges of the whiskers are defined to be 1.5 times the interquartile range (difference between 75th and 25th percentiles).

© 2016 The Authors

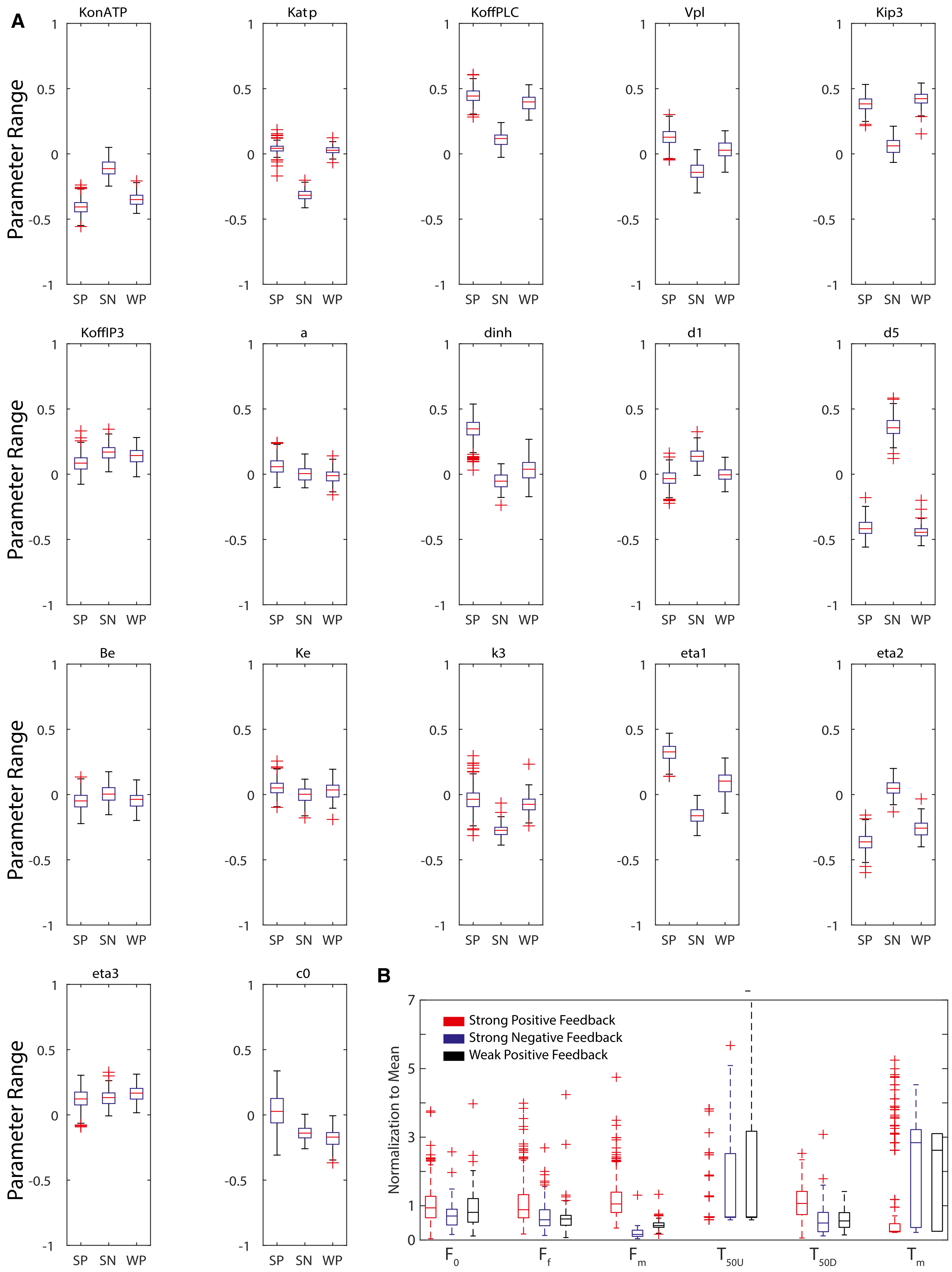

Figure EV4.

 © 2016 The Authors
