# Supplementary material for: Distinct cellular states determine calcium signaling response
Source: Mol Syst Biol. 2016 Dec 15;12(12):894. doi: 10.15252/msb.20167137 (PMC5199124; doi:10.15252/msb.20167137)
Supplement: Supplementary file 2 — Table EV1 [file MSB-12-894-s002.pdf]

Table EV1. Model Parameters

Parameter	Meaning	Value for Center of Prior	Reference
$K_{on,ATP}$ (s^{-1})	Rate constant for PLC generation through ligand binding	1	N/A
K_{ATP} (s^{-1})	Exponential decay constant	8.3e-3	N/A
$K_{off,PLC}$ (s^{-1})	Degradation constant for PLC	1.25	N/A
V_{PLC} ($\mu M/s^{-1}$)	Maximum velocity for IP3 generation	1	N/A
K_{IP3} (µM)	Equilibrium constant for IP3 generation through PLC	0.5	N/A
$K_{off,IP3}$ (s^{-1})	Degradation rate constant of IP3	1.25	N/A
a (s^{-1})	Time constant of IP3 channel	1	Li
d_{inh} (µM)	Dissociation constant for IP3 channel calcium inhibiting subunit	0.4	Li
d1 (µM)	Dissociation constant for IP3 channel IP3 activating subunit	0.13	Lemon
d5(µM)	Dissociation constant For IP3 channel calcium activating subunit	0.0823	Lemon
B_e (µM)	Concentration of calcium buffer	150	Lemon
K_e (µM)	Dissociation constant for calcium buffer	10	Lemon
k3 (µM)	SERCA pump dissociation constant	0.4	Lemon
η_1 (s^{-1})	IP3 channel permeability constant	575	Lemon
η_2 (s^{-1})	ER leak permeability constant	5.2	Lemon
η_3 (s^{-1})	Effective calcium pump permeability	45	Lemon
c_0 (µM)	Total calcium concentration	2	Lemon
